# Effects of dietary N-carbamylglutamate supplementation on growth performance, meat quality, and cecal microbiota in Huoyan geese

**DOI:** 10.3389/fvets.2026.1933899

**Published:** 2026-10-02

**Authors:** Sida Lei, Yirou Xu, Yuchao Zhang, Huacheng Xu, Meiqi Wang, Bin Wang, Di Han, Changjiang Li, Wei Ma, Chunqiang Wang

**Affiliations:** 1 College of Animal Science and Veterinary Medicine, Jinzhou Medical University, Jinzhou, Liaoning, China; 2 Liaoning Provincial Rural and Agricultural Development Service Center, Shenyang, Liaoning, China; 3 Liaoning Modern Agricultural Production Base Construction Engineering Center, Liaoyang, Liaoning, China

**Keywords:** 16S rRNA, cecal microbiota, growth performance, Huoyan geese, meat quality, N-carbamylglutamate

## Abstract

This study evaluated dietary N-carbamylglutamate (NCG) effects on growth performance, body size indices, meat quality, and intestinal morphology in Huoyan geese, aiming to identify the optimal supplemental dose. Only the control and 0.06% NCG groups underwent 16S rRNA sequencing to explore associations between cecal microbiota and meat quality. A total of 240 one-day-old female geese were randomly assigned to four groups, each with six replicates of 10 birds, and fed a basal diet (control group) vs. basal diets supplemented with 0.06, 0.12, and 0.18% NCG groups, respectively, over a 30-week period. Growth performance, body size measurements, and meat quality traits were assessed, and cecal microbial communities were analyzed via 16S rRNA sequencing. From week 6 onward, the 0.06% NCG group exhibited the highest body weight through week 30, with superior average daily gain during the critical 4–8 week growth phase while maintaining normal average daily feed intake. From week 6 through week 30, the 0.06% NCG group had the highest body weight, with superior ADG during weeks 4–8 and while maintaining normal ADFI. Compared with controls, 0.06% NCG significantly increased body oblique length, shank length, and shank circumference (*p* < 0.05), and decreased the feed-to-gain ratio during 1–10 weeks (*p* < 0.05). 0.06%NCG groups also showed significantly increased breast muscle redness (a*) and 24 h postmortem pH (*p* < 0.05), along with decreased shear force in both breast and leg muscles (*p* < 0.05). Jejunal villus height and villus height-to-crypt depth ratio were significantly elevated (*p* < 0.05). 16S rRNA sequencing revealed higher Chao1 and Shannon indices (*p* < 0.05) and altered community structure in the 0.06% group, notably, short-chain fatty acid (SCFA)-producing bacteria, particularly Ruminococcaceae and Faecalibacterium, were enriched in the 0.06% NCG group. Correlation analysis further revealed that these genera were positively correlated with breast muscle redness (a*) and 24 h pH. Dietary 0.06% NCG improved growth performance, body development, and meat quality in Huoyan geese. The beneficial phenotypes may be linked to altered cecal microbiota enriched in SCFA-producing taxa.

## 1 Introduction

The Huoyan goose is an indigenous Chinese waterfowl breed renowned for its high egg production, rapid growth rate, and robust stress resistance. Nevertheless, there exists great potential to further improve its meat quality and feed efficiency (1, 2). In recent years, the use of nutritional regulation strategies to improve poultry productive performance and meat quality has emerged as a research hotspot. N-carbamylglutamate (NCG) is a synthetic structural analogue of endogenous N-acetylglutamate (NAG), the physiological activator of *in vivo* arginine biosynthesis. It can effectively activate carbamoyl phosphate synthetase I (CPS-I) in the mitochondria, thereby promoting arginine synthesis (3). Arginine not only serves as a substrate for protein synthesis but also participates in various physiological processes, including the urea cycle, immune regulation, and vasodilation (4, 5). Previous studies have demonstrated that dietary NCG supplementation can significantly improve growth performance, intestinal barrier function, and antioxidant capacity in weaned piglets (6). In broilers, NCG has been reported to increase average daily gain, improve feed conversion ratio, and enhance intestinal mucosal immune function (7). Furthermore, arginine and its analogues have been found to improve meat quality via the regulation of muscle protein metabolism and fat deposition (8–10). As an exogenous synthetic inducer that upregulates endogenous arginine biosynthesis *in vivo*, NCG optimizes intestinal nutrient supply and strengthens the integrity of the intestinal mucosal barrier, as well as local intestinal immune homeostasis. The improved intestinal microenvironment subsequently alters the community composition and metabolic functionality of cecal microbiota. Collectively, this sequential physiological cascade constructs a potential gut microbiota-dependent signaling axis to explain how NCG supplementation modulates livestock meat quality (11, 12). However, limited information is currently available regarding the application of NCG in geese, and in particular, the interactive relationship among NCG supplementation, cecal microbiota and meat quality has not been systematically characterized.

The cecal microbiota, regarded as the “second genome,” plays a pivotal role in nutritional metabolism, immune regulation, and the formation of meat quality traits (13). Studies have shown that specific microbial populations in the poultry gut, such as short-chain fatty acid (SCFAs)-producing bacteria, can influence muscle pH, color, and tenderness through their metabolites (14–16). Therefore, elucidating whether NCG improves goose meat quality through the modulation of cecal microbiota holds significant theoretical value and practical implications. To this end, Huoyan geese were used to test three graded NCG doses for phenotypic screening of optimal supplementation level. After identifying 0.06% NCG as the optimal dosage based on growth, body size, meat quality and jejunal morphology, we only performed cecal 16S rRNA sequencing on control and 0.06% NCG groups to preliminarily analyze correlations between microbial composition and meat quality. This investigation is expected to provide a scientific basis for the rational application of NCG in goose production.

## 2 Materials and methods

### 2.1 Experimental animals and experimental design

This experiment was approved by the Institutional Animal Care and Use Committee of Jinzhou Medical University (Approval No. JZMULL2026131) and was conducted in compliance with the EU Directive 2010/63/EU and the 3R principles. The trial was carried out at the National Huoyan Goose Conservation Farm of China (Registry No. C2111501).

A total of 240 one-day-old female Huoyan geese with an initial body weight of 77.61 ± 6.60 g were selected. Following a 7-day acclimation period, the birds were randomly assigned to four groups using a completely randomized single-factor design, with six replicates per group and ten birds per replicate. The control group received a basal diet, while the three experimental groups were fed the basal diet supplemented with 0.06, 0.12, and 0.18% NCG, respectively, over a 30-week experimental period. The optimal supplemental dosage was screened based on growth performance indicators, and this dosage was adopted for subsequent microbiota and meat quality analyses.

Geese were raised in a slatted-floor housing system. The stocking density was 6 geese/m^2^ from 1 to 4 weeks of age, and subsequently adjusted to 3 geese/m^2^ after 6 weeks of age. Temperature was maintained at 32–34 °C from 1 to 3 days of age, 30–32 °C from 4 to 7 days of age, 27–29 °C from 2 to 3 weeks, 24–26 °C from 4 to 5 weeks, and 21–23 °C after 6 weeks of age. Relative humidity was kept at 60–70%. All geese were provided with ad libitum access to experimental diets and clean fresh drinking water throughout the whole experimental period. Feed was added twice daily at 08:00 and 17:00 to guarantee continuous feed supply in the trough. For the lighting program, a 24 h continuous artificial light was applied during the first week for brooding adaptation. From week 2 to week 16, the light schedule was set as 16 h light: 8 h dark. After 16 weeks of age, natural daylight was adopted with proper shading to avoid excessive light stimulation, which conformed to the conventional feeding management of Huoyan geese. Regular pen cleaning and disinfection were performed to maintain sanitary conditions during the trial. Each pen was equipped with a swimming pond (8 m × 3 m × 1.3 m). Feces and drinking facilities were cleaned and disinfected on a daily basis. All birds were vaccinated according to conventional procedures. The NCG product (purity ≥ 99%) was provided by Yuanchang Industrial Co., Ltd., Jiangxi, China. The basal diet was formulated referring to *Feeding Standard of China for Geese* (31st Edition, 2020), and its ingredient composition and nutrient levels are presented in Table 1.

**Table 1:** Composition and nutrient levels of the basal diet (air-dry basis, %).

Stage	Starter	Grower	Developer	Stage	Starter	Grower	Developer
Ingredient	Content	Content	Content	Nutrient levels	Content	Content	Content
Corn	55	59.72	62.72	Metabolizable energy (MJ/kg)	12.03	12.20	12.28
Soybeanmeal	25	20	16	Crude protein	18.00	16.01	14.70
Wheat bran	8	7	7	Crude fiber	3.42	3.17	3.13
Corn fermented feed	7.12	8.4	10	Calcium	0.87	0.89	0.72
Sodium chloride	0.35	0.35	0.35	Total phosphorus	0.63	0.59	0.51
Limestone	1.8	1.8	1.4	Lysine	0.94	0.82	0.74
Dicalcium phosphate	1.2	1.2	1	Methionine	0.42	0.39	0.37
Lysine	0.1	0.1	0.1				
Methionine	0.18	0.18	0.18				
Vitamin-mineral premix	1.25	1.25	1.25				
Total	100	100	100				

The premix provided the following per kilogram of the diet: vitamin A 5,000 IU, vitamin D₃ 1,000 IU, vitamin E 30 mg, vitamin B₁ 3 mg, vitamin B₂ 5 mg, vitamin K₃ 4 mg, vitamin B₆ 3 mg, pantothenic acid 18 mg, nicotinic acid 65 mg, biotin 0.1 mg, folic acid 2.0 mg, vitamin B₁₂ 0.025 mg, Fe 80 mg, Zn 85 mg, Mn 80 mg, Cu 6 mg, Se 0.3 mg, I 0.45 mg, and Co 2.5 mg.

### 2.2 Growth performance measurement

Body weights were measured after a 12 h overnight fast at 2-week intervals during the first 10 weeks (70 days) of the experimental period, and thereafter at 4-week intervals until the end of the trial at 30 weeks (210 days). Average daily feed intake (ADFI), average daily gain (ADG), and feed-to-gain ratio (F/G) were calculated as follows:

ADFI=Total feed intake/Experimental days

$$\begin{array}{l} \mathrm{ADG}=(\text{Final body weight}–\text{Initial body weight})/\\ \text{Experimental days} \end{array}$$

F/G=Total feed intake/Total body weight gain

### 2.3 Body size index measurement

Body size indices were measured in accordance with the *Terms and Measurement Statistical Methods for Poultry Production Performance* (NY/T 823-2020). At 60 days of age, one goose was randomly selected from each replicate for body size measurements. The measured body size indices included body oblique length, Semi-diving length, neck length, keel length, shank length, shank circumference, breast depth, breast width, and hip width.

### 2.4 Sample collection

At the conclusion of the experimental period, six geese were randomly selected from each group after a 12 h fast. After body weight measurement, the geese were euthanized via intravenous injection of 1 mL of Euthasol.

The entire intestinal tract was dissected from the proventriculus to the cloaca. Cecal contents were collected into cryogenic vials and immediately preserved in liquid nitrogen for subsequent 16S rRNA sequencing. Following this, the intestinal contents were gently rinsed with physiological saline. The jejunal segment was isolated, weighed, and its length was measured. Subsequently, an approximately 1 cm segment from the middle portion of the jejunum was collected and placed in 4% neutral buffered formalin fixative solution for histological sectioning to measure villus height, crypt depth, and related parameters.

Samples were taken from the central part of the right pectoralis major for breast muscle, and the middle section of the right femoral biceps for thigh muscle to determine meat color, pH, shear force, and water-holding capacity.

### 2.5 Intestinal morphology determination

The fixed intestinal tissue samples (jejunum) were processed through trimming, washing, dehydration, clearing, paraffin embedding, trimming of paraffin blocks, and sectioning. The sections were then stained with hematoxylin–eosin (HE) and examined under an inverted optical microscope equipped with a video imaging system (Olympus, Japan). Villus height and crypt depth were measured, and the villus height-to-crypt depth ratio (V/C ratio) was subsequently calculated.

### 2.6 Meat quality determination

Meat color was measured using a Konica Minolta CR-400 colorimeter (Tokyo, Japan); the lightness (L*), redness (a*), and yellowness (b*) values of breast and leg muscles were recorded at 45 min and 24 h postmortem. The pH of breast and leg muscles at 45 min and 24 h postmortem was measured using a calibrated HANNA HI99163 pH meter (Italy). For shear force measurement, muscle strips were cut parallel to the orientation of muscle fibers with a uniform dimension of 1.0 cm × 1.0 cm × 3.0 cm. Shear force was determined using a Stable Micro Systems TA XTplus texture analyzer (UK) equipped with a Warner-Bratzler shear probe, and the maximum peak shear force was recorded with six repeated tests per sample. The water-holding capacity was determined by the pressure method. Briefly, approximately 1.0 g fresh muscle sample was wrapped with quantitative filter paper, placed on a flat pressure platform, and subjected to a constant pressure of 35 kg for 5 min. After pressure release, the wet area filter paper was weighed, and the water loss rate was calculated to characterize water-holding capacity. Supplementary texture parameter detection of water-holding capacity was performed using a Stable Micro Systems TA XT2i texture analyzer (UK).

### 2.7 Intestinal microbial diversity analysis

Based on the results of NCG supplementation on growth performance, body size indices, and meat quality in geese, the 0.06% NCG group and the control group were selected for cecal microbiota community structure analysis.

Total DNA was extracted from cecal contents of Huoyan geese using the CTAB-SDS method. DNA purity and concentration were assessed using a Nanodrop One spectrophotometer. The V4 hypervariable region of the 16S rRNA gene was amplified using the barcoded specific primers 515F (5′-GTGYCAGCMGCCGCGGTAA-3′) and 806R (5′-GGACTACHVGGGTWTCTAAT-3′), and sequencing was performed on the Illumina NovaSeq 6000 platform. Raw sequencing reads were subjected to quality control using Fastp software (v0.14.1), and paired-end reads were assembled using the fastq_mergepairs module of usearch (v10) based on overlap characteristics. Operational taxonomic units (OTUs) were clustered at a 97% similarity threshold, and chimeric sequences were removed. Taxonomic annotation was performed against the SILVA database with a confidence threshold of ≥0.8 (17). Alpha diversity was assessed using the Chao1 index, Shannon index, and Simpson index, while beta diversity was evaluated through principal coordinate analysis (PCoA) and non-metric multidimensional scaling (NMDS).

### 2.8 Statistical analysis

Data on growth performance and intestinal morphology were statistically analyzed and graphically visualized using GraphPad Prism 10.0 software (GraphPad Software, San Diego, CA, United States). One-way analysis of variance (ANOVA) was performed, and significant differences among groups were annotated using compact letter display (CLD). Body size indices and meat quality parameters were analyzed using SPSS 27.0 software. One-way ANOVA was performed, followed by Duncan’s multiple range test for post-hoc comparisons. All data were expressed as mean ± standard deviation (SD), and differences were considered statistically significant at *p* < 0.05. Correlation analysis between the relative abundance of cecal microbiota at the phylum level and meat quality traits was performed and visualized using R software (version 4.5.2). Spearman’s correlation coefficients and their significance were calculated, and the direction, significance, and magnitude of correlations were presented using an integrated heatmap combined with correlation link lines.

## 3 Results

### 3.1 Effects of dietary NCG supplementation on growth performance of Huoyan geese

The body weights of Huoyan geese in all groups followed the typical S-shaped growth curve characteristic of poultry. A rapid growth phase was observed from 1 day to 14 weeks of age, followed by a growth plateau phase after 14 weeks, with body weights stabilizing at 30 weeks of age. After 6 weeks of age, the 0.06% NCG group exhibited the highest body weight among all groups, surpassing the control group as well as the 0.12 and 0.18% NCG groups. After 22 weeks of age, body weight in the 0.18% NCG group was lower than that in the control, 0.06% NCG, and 0.12% NCG groups (Figure 1A).

**Figure 1:**
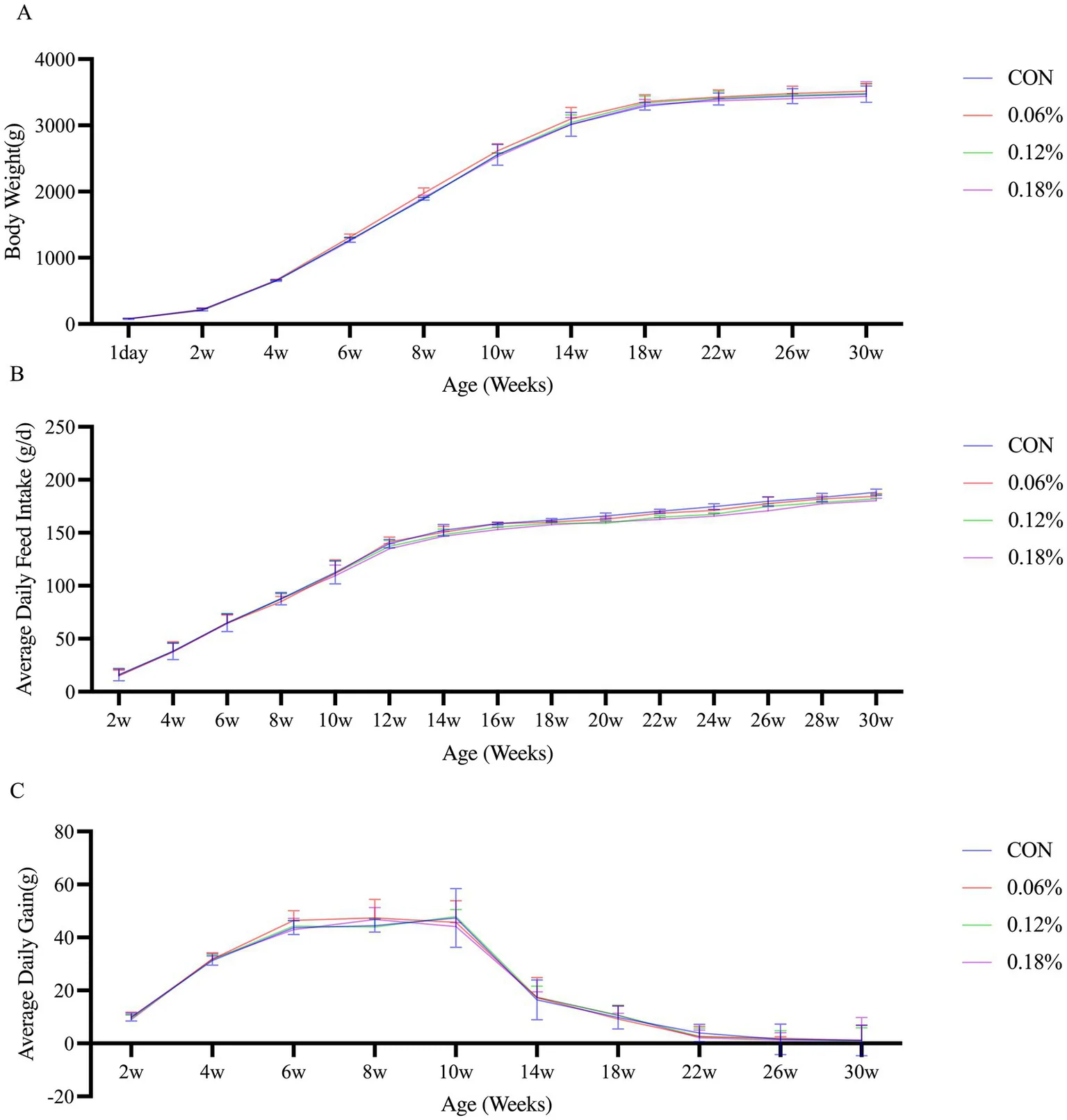
Effects of dietary NCG supplementation on growth performance of Huoyan geese. **(A)** Body weight growth curve; **(B)** Average daily feed intake (ADFI); **(C)** Average daily gain (ADG). CON stands for the blank control group; 0.06, 0.12 and 0.18% represent the treatment groups supplemented with the corresponding doses of NCG in basal diet, respectively.

Daily feed intake in all groups exhibited a continuous upward trend with increasing age, with a rapid increase from 2 to 12 weeks of age, after which the growth rate slowed and stabilized. After 8 weeks of age, the 0.18% NCG group consistently showed lower daily feed intake than the control, 0.06% NCG, and 0.12% NCG groups, while the control group maintained the highest feed intake throughout the experimental period (Figure 1B).

Average daily gain in all groups exhibited a pattern of initial increase followed by a subsequent decline. The rapid growth phase occurred from 2 to 10 weeks of age, with ADG increasing rapidly from 2 to 6 weeks and reaching its peak between 6 and 10 weeks of age. After 10 weeks of age, ADG gradually declined and approached zero after 22 weeks of age. Notably, the 4- to 8-week period was identified as the critical phase for weight gain, during which the 0.06% NCG group demonstrated superior ADG compared with the control, 0.12% NCG, and 0.18% NCG groups (Figure 1C).

During the 1–10 weeks of age, the F/G ratio in the 0.06% NCG group was significantly lower than that in the control group (*p* < 0.05), whereas no significant differences were observed among groups during the 10–30 weeks period (*p* > 0.05). Notably, the F/G ratio in the 0.12% NCG group was significantly lower than that in the control group over the entire experimental period (*p* < 0.05) (Figures 2A–C).

**Figure 2:**
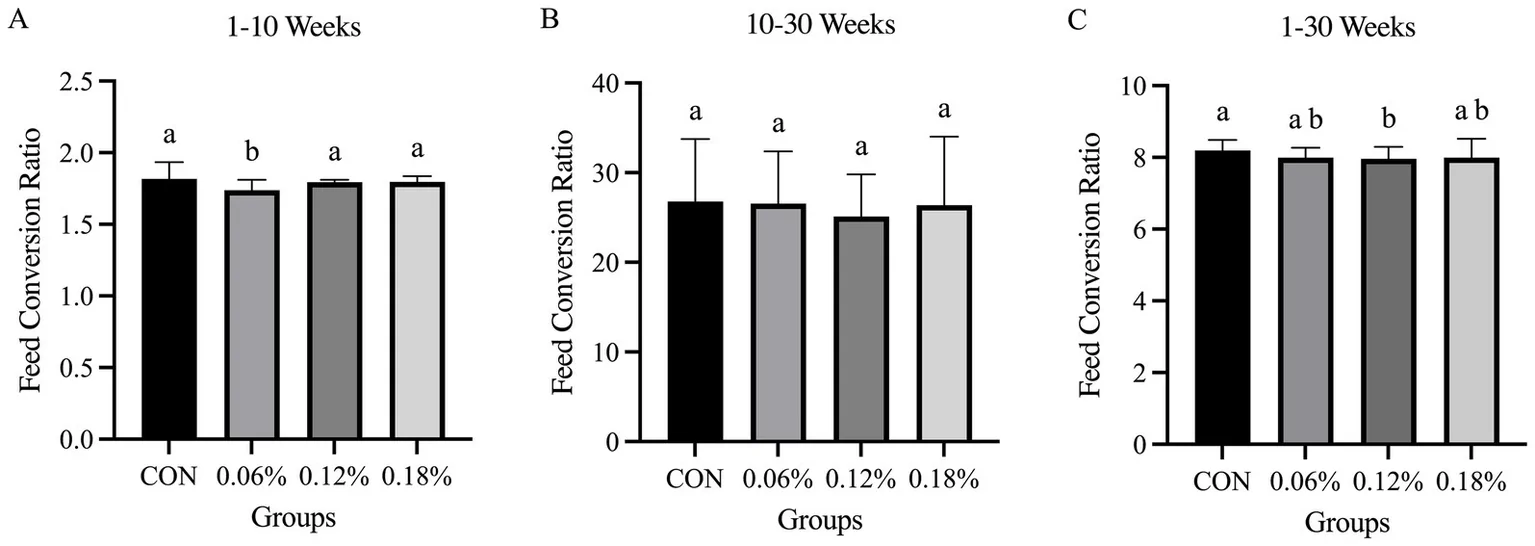
Effects of dietary NCG supplementation on phase feed-to-gain ratio of Huoyan geese. **(A)** Feed-to-gain ratio at 1–10 weeks of age; **(B)** Feed-to-gain ratio at 10–30 weeks of age; **(C)** Overall feed-to-gain ratio for the whole 1–30-week experimental period. CON stands for the blank control group; the 0.06, 0.12 and 0.18% groups are the treatment groups supplemented with corresponding doses of NCG in basal diet, respectively. Different lowercase letters above bars indicate significant differences among groups (*p* < 0.05); identical letters or the absence of letters mean no significant difference (*p* > 0.05). All data are presented as mean ± standard deviation (SD).

### 3.2 Effects of dietary NCG supplementation on body size indices of Huoyan geese

As shown in Table 2, compared with the control group, dietary supplementation with 0.06% NCG significantly increased body oblique length, shank length, and shank circumference (*p* < 0.05). Neck length also exhibited an increasing trend, although the difference did not reach statistical significance (*p* > 0.05). No significant differences were observed among the supplementation groups and the control group in terms of Semi-diving length, keel length, breast depth, breast width, or hip width (*p* > 0.05).

**Table 2:** The effects of NCG on body measurements of Huoyan geese.

Item	CON	0.06%	0.12%	0.18%	*P*
Body oblique length	22.18 ± 0.63^bc^	23.47 ± 0.65^a^	23.00 ± 0.54^ab^	22.03 ± 1.07^c^	0.01
Semi-diving length	55.50 ± 4.64	57.33 ± 2.07	56.83 ± 2.93	58.50 ± 1.52	0.41
Neck length	26.00 ± 1.90	28.67 ± 2.42	25.75 ± 1.60	26.00 ± 1.79	0.06
Keel length	14.92 ± 0.60^ab^	15.35 ± 0.52^a^	14.70 ± 0.54^b^	14.50 ± 0.18^b^	0.04
Shank length	7.45 ± 0.24^b^	7.90 ± 0.17^a^	7.53 ± 0.29^b^	7.58 ± 0.27^b^	0.03
Shank circumference	4.58 ± 0.35^b^	4.97 ± 0.18^a^	4.67 ± 0.14^b^	4.62 ± 0.17^b^	0.03
Breast depth	7.42 ± 0.15	7.50 ± 0.14	7.30 ± 0.29	7.22 ± 0.25	0.15
Breast width	6.33 ± 0.34	6.77 ± 0.37	6.55 ± 0.58	6.62 ± 0.40	0.40
Hip width	7.17 ± 0.23	7.27 ± 0.50	7.23 ± 0.26	7.27 ± 0.35	0.96

Within the same row, values with different lowercase superscripts indicate significant differences (*p* < 0.05), while those with the same or no superscripts indicate no significant differences (*p* > 0.05).

### 3.3 Effects of dietary NCG supplementation on meat quality of Huoyan geese

As presented in Tables 3, 4, compared with the control group, at 45 min postmortem, no significant differences were observed in meat color parameters (L*, a*, b*) of breast and leg muscles across all NCG supplementation groups, with the exception that breast muscle redness (a*) in the 0.06% NCG group exhibited a numerical increasing trend. Compared with the control group, at 24 h postmortem, breast muscle redness (a*) was significantly increased in the 0.06, 0.12, and 0.18% NCG groups (*p* < 0.05). Although lightness (L*) and yellowness (b*) showed certain trends of change, the differences did not reach statistical significance (*p* > 0.05). No significant differences were observed among groups in any color parameters of leg muscle (*p* > 0.05).

**Table 3:** Effects of NCG on breast muscle meat quality of Huoyan geese.

Item	Con	0.06%	0.12%	0.18%	*P*
Lightness (L*) _45min_	39.56 ± 4.05	38.74 ± 2.17	38.89 ± 1.12	38.99 ± 2.22	0.95
Redness (a*) _45min_	10.53 ± 0.86	10.75 ± 0.83	11.15 ± 0.32	11.10 ± 0.70	0.39
Yellowness (b*) _45min_	9.23 ± 0.54	9.28 ± 0.50	9.50 ± 0.31	9.45 ± 0.23	0.63
Lightness (L*) _24h_	42.87 ± 0.95	41.79 ± 0.44	41.83 ± 0.89	41.78 ± 0.86	0.08
Redness (a*) _24h_	9.24 ± 0.60^b^	10.50 ± 0.62^a^	10.18 ± 0.48^a^	10.16 ± 0.95^a^	0.03
Yellowness (b*) _24h_	10.85 ± 1.00	10.36 ± 0.71	11.40 ± 0.69	11.45 ± 0.54	0.07
muscle pH_45min_	6.79 ± 0.08	6.82 ± 0.04	6.81 ± 0.05	6.78 ± 0.03	0.58
muscle pH_24h_	5.76 ± 0.32^b^	6.17 ± 0.18^a^	6.01 ± 0.23^ab^	5.89 ± 0.12^b^	0.03
Shear force (N)	44.82 ± 7.76^b^	39.53 ± 4.30^a^	41.24 ± 5.55^ab^	42.06 ± 4.40^ab^	0.05
Water-holding capacity (%)	23.35 ± 1.43	22.96 ± 2.05	23.97 ± 1.65	23.92 ± 1.24	0.67

Within the same row, values with different lowercase superscripts indicate significant differences (*p* < 0.05), while those with the same or no superscripts indicate no significant differences (*p* > 0.05).

**Table 4:** Effects of NCG on leg muscle meat quality of Huoyan geese.

Item	Con	0.06%	0.12%	0.18%	*P*
Lightness (L*) _45min_	41.25 ± 1.94	42.39 ± 0.78	41.68 ± 1.19	41.80 ± 1.70	0.62
Redness (a*) _45min_	11.63 ± 1.40	12.04 ± 1.02	12.23 ± 0.87	12.36 ± 1.14	0.70
Yellowness (b*) _45min_	11.80 ± 1.45	10.49 ± 1.28	11.48 ± 1.30	11.90 ± 0.95	0.23
Lightness (L*) _24h_	44.03 ± 2.08	44.74 ± 1.40	44.25 ± 1.24	43.29 ± 1.64	0.49
Redness (a*) _24h_	10.48 ± 0.46	11.78 ± 1.12	11.14 ± 1.43	11.43 ± 1.32	0.28
Yellowness (b*) _24h_	12.99 ± 1.29	11.51 ± 1.32	12.26 ± 1.73	12.05 ± 1.13	0.35
muscle pH_45min_	6.81 ± 0.06	6.81 ± 0.03	6.78 ± 0.03	6.77 ± 0.06	0.31
muscle pH_24h_	6.05 ± 0.24	6.36 ± 0.21	6.19 ± 0.07	6.23 ± 0.19	0.07
Shear force (N)	55.88 ± 4.73^c^	51.39 ± 4.12^a^	53.13 ± 6.35^ab^	54.03 ± 3.19^ab^	0.05
Water-holding capacity (%)	25.08 ± 2.33	23.65 ± 1.88	23.37 ± 1.70	24.38 ± 1.77	0.43

Within the same row, values with different lowercase superscripts indicate significant differences (*p* < 0.05), while those with the same or no superscripts indicate no significant differences (*p* > 0.05).

Compared with the control group, no significant differences were observed in the pH values of breast or leg muscles across all NCG-supplemented groups at 45 min postmortem (*p* > 0.05). At 24 h postmortem, the 0.06% NCG group exhibited a significantly higher pH value in breast muscle compared with the control group (*p* < 0.05), while the 0.12 and 0.18% NCG groups showed increasing trends that did not reach statistical significance (*p* > 0.05). Regarding leg muscle pH, the 0.06% NCG group was significantly higher than the control group (*p* < 0.05), whereas the 0.12 and 0.18% NCG groups exhibited upward trends without significant differences (*p* > 0.05).

Compared with the control group, shear force values of both breast and leg muscles were significantly decreased in the 0.06% NCG group (*p* < 0.05), while the 0.12 and 0.18% NCG groups showed decreasing trends that did not reach statistical significance (*p* > 0.05).

Compared with the control group, the percentage of water loss in both breast and leg muscles exhibited decreasing trends across all NCG-supplemented groups, although no significant differences were observed among groups (*p* > 0.05).

### 3.4 Effects of dietary NCG supplementation on intestinal morphology of Huoyan geese

Representative H&E-stained jejunal micrographs of all four groups are presented in Figures 3A–D to visually display mucosal morphological differences. Compared with the control group, dietary supplementation with 0.06% NCG significantly increased villus height (VH) (*p* < 0.05). No significant effects on crypt depth (CD) were observed across all supplementation groups (*p* > 0.05). Furthermore, the 0.06 and 0.12% NCG supplementation groups exhibited significantly increased villus height-to-crypt depth ratios (VH/CD ratio) (*p* < 0.05) (Figure 3).

**Figure 3:**
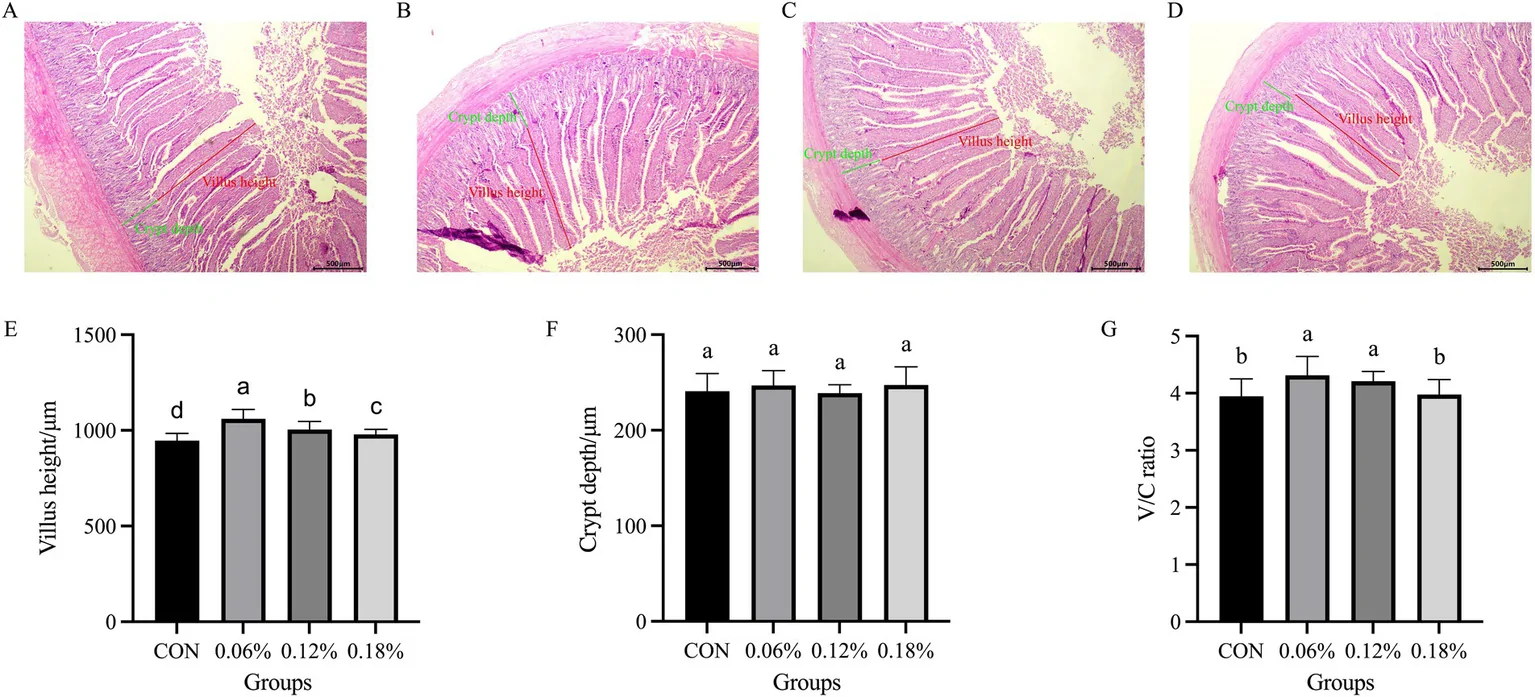
Effects of dietary NCG supplementation on intestinal morphology of Huoyan geese. **(A)** Villus height (unit: μm); **(B)** Crypt depth (unit: μm); **(C)** Villus height-to-crypt depth ratio (V/C, dimensionless). CON stands for the blank control group; the 0.06, 0.12 and 0.18% groups are treatment groups supplemented with corresponding doses of NCG in basal diet, respectively. Different lowercase letters above bars indicate significant differences among groups (*p* < 0.05); identical letters or the absence of letters mean no significant difference (*p* > 0.05). All data are presented as mean ± standard deviation (SD). Panels **A–D** show HE-stained intestinal tissue sections to present histological morphology. Panels **E–G** display Prism-based one-way ANOVA results, including histograms of intestinal villus height, crypt depth, and villus/crypt ratio for quantitative morphological comparison among groups.

### 3.5 Effects of dietary NCG supplementation on cecal microbiota of Huoyan geese

Following integrated assessment of growth performance, jejunal histological morphology and meat quality traits, the 0.06% NCG treatment group with the most favorable comprehensive phenotypes was subjected to comparative analysis of cecal microbial community composition.

#### 3.5.1 OTU statistics and diversity analysis

The length distribution histogram of OTU sequences (Figure 4A) revealed that the lengths of all clustered OTU sequences were highly concentrated within the 400–420 bp interval, with the peak sequence length occurring at 400 bp, where the corresponding OTU count was significantly higher than in other intervals. In the 300–390 bp range, almost no valid OTUs were detected, with only a few scattered low-abundance sequences.

**Figure 4:**
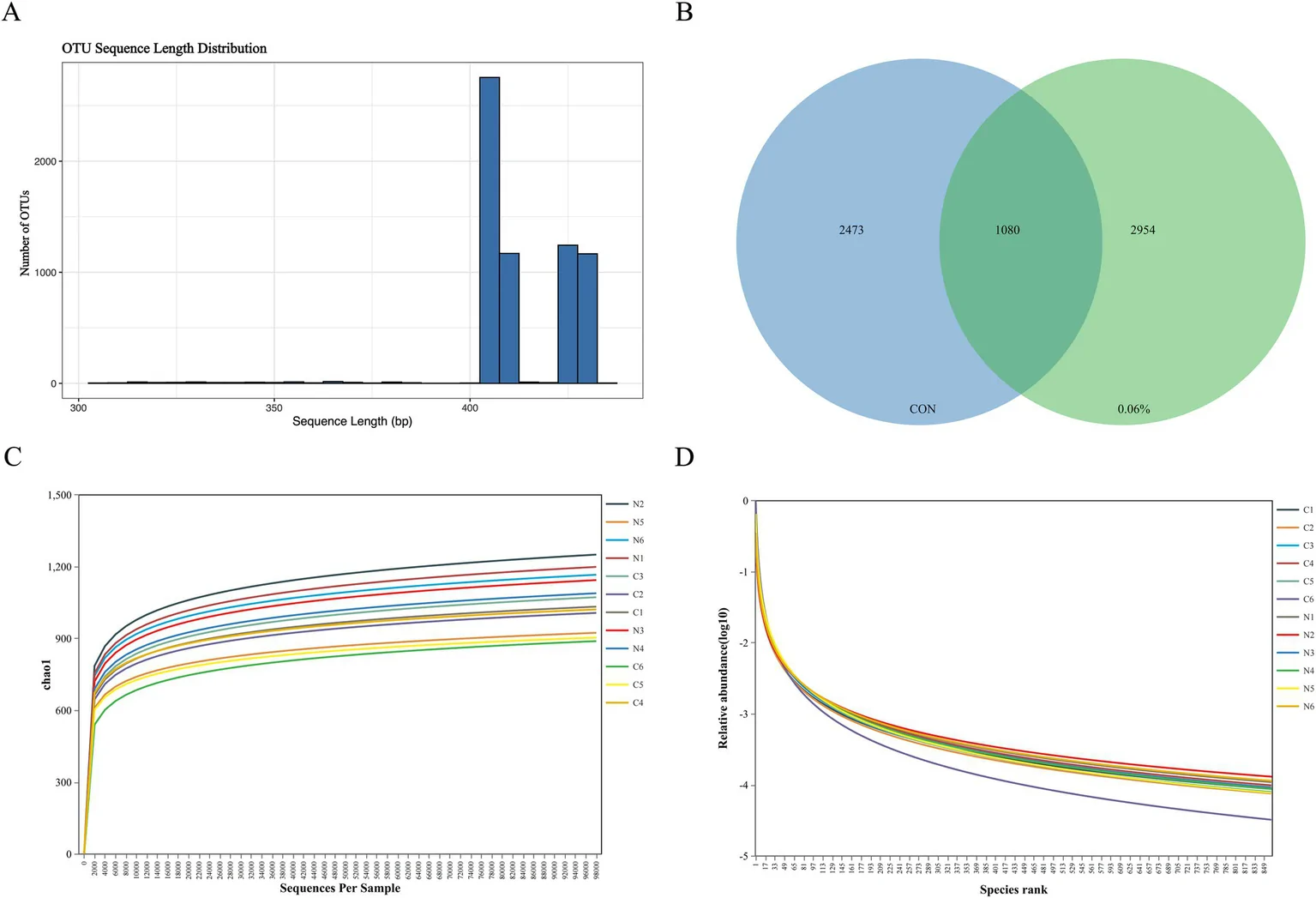
Effects of dietary NCG supplementation on cecal microbiota of Huoyan geese. Panel **(A)** shows the length distribution of OTU sequences, which reflects the distribution characteristics of sequence lengths of all OTUs. Panel **(B)** is a Venn diagram displaying the numbers of shared and unique OTUs of intestinal microbiota between the CON group and the 0.06% NCG group. Panel **(C)** presents the Rank-Abundance curve, which reflects the species abundance distribution and evenness within samples. Panel **(D)** shows the Chao1 rarefaction curve for evaluating the coverage of species diversity under different sequencing depths. CON represents the blank control group, and 0.06% stands for the NCG supplementation group.

Venn diagram analysis (Figure 4B) showed that a total of 6,507 OTUs were obtained from the control group (CON) and the 0.06% NCG group. Among these, the control group had 2,473 unique OTUs, accounting for 38.0% of the total OTUs, while the 0.06% NCG group had 2,954 unique OTUs, representing 45.4% of the total OTUs. The two groups shared 1,080 common OTUs, accounting for 16.6% of the total OTUs. The rarefaction curves (Figure 4C) showed that the OTU counts of all samples gradually approached a plateau, indicating that the sequencing depth was sufficient to cover the majority of microorganisms in the samples. Notably, the microbial species richness of each sample in the 0.06% NCG group was higher than that of the control group samples. The rank-abundance curves (Figure 4D) further demonstrated that the curves of the 0.06% NCG group samples were smoother, suggesting greater species evenness in the microbial community of this group.

The OTU-based PCoA (Figure 5A) and genus-level PCoA (Figure 5C) results revealed that the control group samples were relatively scattered in the plot, whereas the 0.06% NCG group samples were tightly clustered on the right side of the plot, showing a clear separation trend between the two groups. This indicated that 0.06% NCG supplementation significantly altered the cecal microbial community structure and enhanced the intra-group stability of the microbiota. However, the phylum-level PCoA (Figure 5B) showed that the confidence intervals of the control and 0.06% NCG groups largely overlapped without obvious separation, suggesting that no significant differences in cecal microbial community structure existed between the two groups at the phylum level, which is consistent with the stable abundances of the core dominant phyla.

**Figure 5:**
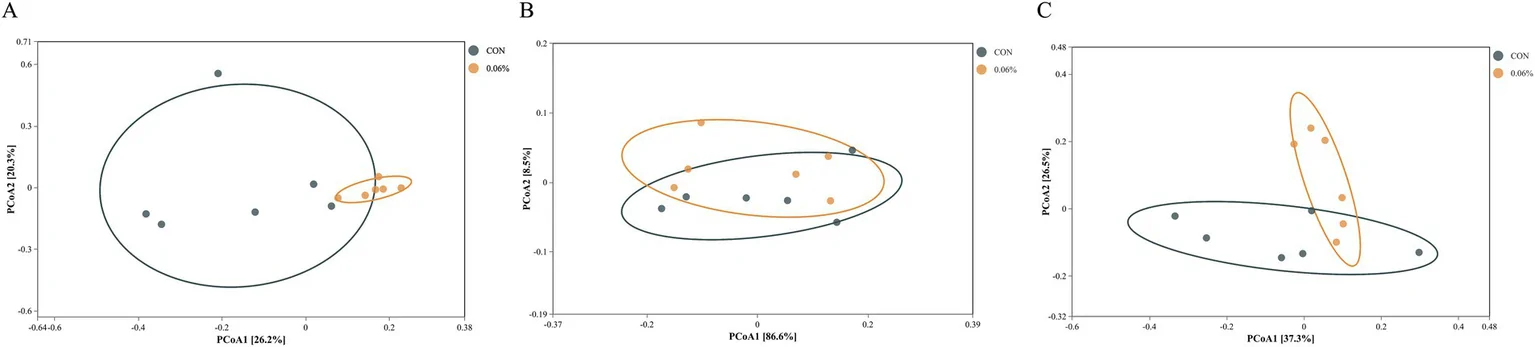
Effects of dietary NCG supplementation on PCoA of cecal microbiota in Huoyan geese. Panels **(A–C)** show PCoA plots based on Bray-Curtis distance at the OTU, phylum and genus levels, respectively. CON refers to the blank control group, and 0.06% stands for the NCG supplementation group. Ellipses represent the 95% confidence interval.

#### 3.5.2 Cecal microbial community structure analysis

The phylum-level community composition results (Figure 6A) showed that the cecal microbiota in both the control group and the 0.06% NCG group were predominated by Firmicutes and Bacteroidota as the core dominant phyla, with their combined relative abundances accounting for more than 85% of the total community. Specifically, Firmicutes accounted for 58.59% in the control group and 56.73% in the 0.06% NCG group, while Bacteroidota accounted for 32.98% in the control group and 31.19% in the 0.06% NCG group, with no substantial differences observed between the two groups. All low-abundance phyla (Proteobacteria, Desulfobacterota, Deferribacterota, Fusobacteriota, and Verrucomicrobiota) had average relative abundances below 3% in both groups. The average proportion of Bacteroidota was slightly higher in the control group (2.57%) than in the 0.06% NCG group (2.13%). Distinct intergroup shifts were detected among the remaining low-abundance phyla: Fusobacteriota and Desulfobacterota were markedly reduced from their respective baseline proportions (1.69% for Fusobacteriota) in the control group to lower levels (0.40% for Fusobacteriota) in the 0.06% NCG group, while Verrucomicrobiota and Deferribacterota were markedly enriched. Unclassified taxa (pooled as “others”) showed a mild upward trend after NCG supplementation (0.93% in the control group vs. 1.36% in the 0.06% NCG group).

**Figure 6:**
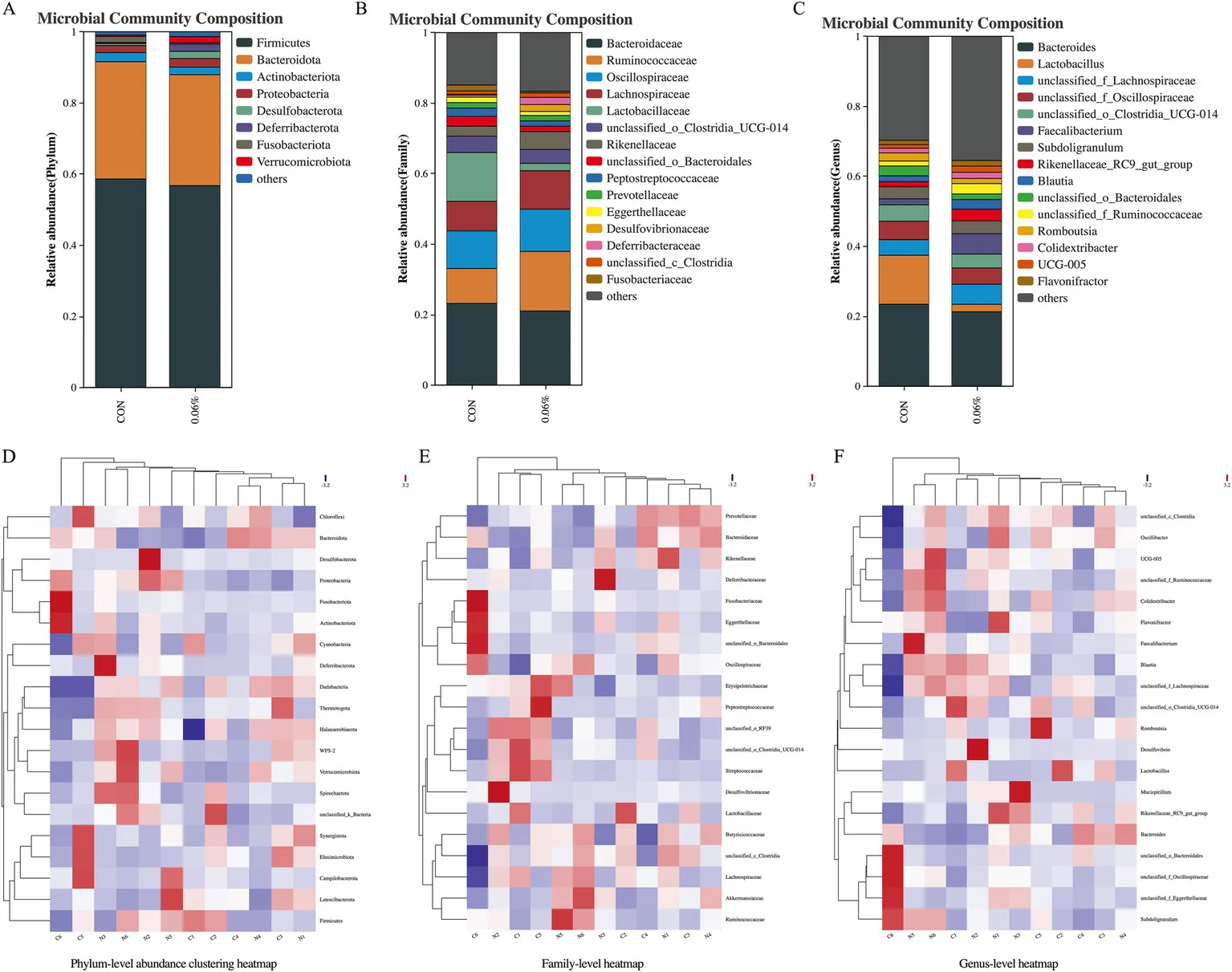
Analysis of cecal microbial community structure in Huoyan geese fed diets supplemented with NCG. Panels **(A–C)** show stacked bar charts of the relative abundance of intestinal microbiota at the phylum, family and genus levels, respectively; Panels **(D–F)** display clustering heatmaps of microbial abundance at phylum, family and genus levels. CON represents the blank control group, and 0.06% stands for the NCG supplementation group.

The phylum-level species abundance clustering heatmap (Figure 6D) revealed a clear separation in the overall cecal microbial community structure between the control group and the 0.06% NCG (N2) group. At the individual sample level, some samples in the 0.06% NCG group exhibited higher abundances of Firmicutes; however, the group mean results from the stacked bar chart (Figure 6A) showed that the overall average abundance of Firmicutes in the 0.06% NCG group was slightly lower than that in the control group. Regarding potentially pathogenic phyla, the abundances of Fusobacteriota and Actinobacteriota were generally lower in the 0.06% NCG group samples than in the control group samples. Bacteroidota was a core dominant phylum shared by both groups, with no obvious difference observed between groups; other low-abundance phyla, such as Chloroflexi and Cyanobacteria, did not exhibit consistent patterns of change between groups.

Family-level community composition results from the stacked bar chart (Figure 6B) showed that the cecal microbiota in both the control group and the 0.06% NCG treatment group had Bacteroidaceae as the core dominant family. Specifically, Bacteroidaceae accounted for 23.15% in the control group and 20.99% in the 0.06% NCG group, with only a slight difference in average relative abundance between the two groups. The relative proportion of Lactobacillaceae reached 13.86% in the control group, evidently higher than that in the 0.06% NCG group, which showed the most distinct intergroup variation in relative abundance among all detected families. SCFAs-producing families, including Ruminococcaceae, Oscillospiraceae and Lachnospiraceae, displayed higher average relative abundances in the 0.06% NCG group, with Ruminococcaceae presenting the largest visual upward shift. For minor low-abundance families, Prevotellaceae exhibited nearly identical relative abundances between groups, while Eggerthellaceae had a mild reduction in the NCG group; Fusobacteriaceae, however, decreased markedly after NCG supplementation. Unclassified taxa (pooled as “others”) showed a mild upward trend after NCG supplementation (14.86% in the control group vs. 16.68% in the 0.06% NCG group). implying a diverse pool of low-abundance rare taxa within the cecal microbial ecosystem.

The family-level abundance clustering heatmap (Figure 6E) further illustrated the inter-individual variation of cecal microbiota among replicate samples. Compared with samples from the control group, the representative N2 replicate of the 0.06% NCG group possessed markedly higher relative abundances of SCFA-producing families, represented by Ruminococcaceae. In contrast, Fusobacteriaceae, Eggerthellaceae and unclassified_o_Bacteroidales all showed generally lower relative abundances in the N2 sample. Bacteroidaceae, the dominant core family shared by both groups, exhibited negligible intergroup differences. The rest low-abundance families failed to show consistent abundance alteration trends across replicates between the two treatments.

Genus-level community composition results from the stacked bar chart (Figure 6C) revealed that *Bacteroides* was the core dominant genus in both the control group and the 0.06% NCG treatment group, accounting for 23.41 and 21.26% of the total cecal community, respectively, with no substantial intergroup difference in average relative abundance. The average proportion of *Lactobacillus* reached 14.01% in the control group and 2.14% in the 0.06% NCG group, evidently higher in the control treatment; this genus showed the most distinct intergroup variation among all detected genera. Unclassified_f_*Lachnospiraceae* displayed slightly higher average abundance in the control group, whereas SCFA-producing genera including unclassified_f_*Ruminococcaceae* and *Faecalibacterium* exhibited markedly higher average abundances in the 0.06% NCG group, while *Romboutsia* was reduced after NCG supplementation. Other low-abundance genera including *Blautia*, *Flavonifractor*, *Subdoligranulum*, and UCG-005 all presented elevated relative abundances in the NCG group, with varying degrees of intergroup difference. Unclassified taxa (pooled as “others”) showed a mild upward trend after NCG supplementation (29.71% in the control group vs. 35.46% in the 0.06% NCG group). respectively, implying a diverse pool of low-abundance rare taxa within the cecal microbial ecosystem.

The genus-level abundance clustering heatmap (Figure 6F) further visualized the discrete variations in relative abundance of cecal microbiota across individual replicate samples. Compared with control replicates, the representative N2 sample of the 0.06% NCG group had markedly higher relative abundances of SCFA-producing genera, such as unclassified_f_*Ruminococcaceae* and *Faecalibacterium*. In contrast, *Lactobacillus*, unclassified_o_*Bacteroidales* and unclassified_f_*Lachnospiraceae* all exhibited generally lower relative abundances in the N2 sample. *Bacteroides*, the dominant core genus shared by both groups, showed negligible intergroup differences. The other low-abundance genera failed to display consistent abundance alteration trends across all replicate samples between the two treatments.

#### 3.5.3 Differential analysis of cecal microbiota

The linear discriminant analysis effect size (LEfSe) bar plot (Figure 7A) identified statistically differential taxa of cecal microbiota between the control group and the 0.06% NCG group. Only class *Bacilli* (predominantly affiliated to genus *Lactobacillus*) was significantly enriched in the control group, with an LDA score of 4.0. In contrast, the 0.06% NCG group harbored abundant differentially enriched taxa across multiple taxonomic ranks. The core functional taxa producing short-chain fatty acids spanned order *Oscillospirales*, family *Ruminococcaceae*, and genus *Faecalibacterium*, most of which had LDA scores above 3.0, and several taxa reached LDA scores close to 6.0, reflecting a strong enrichment effect under NCG supplementation.

**Figure 7:**
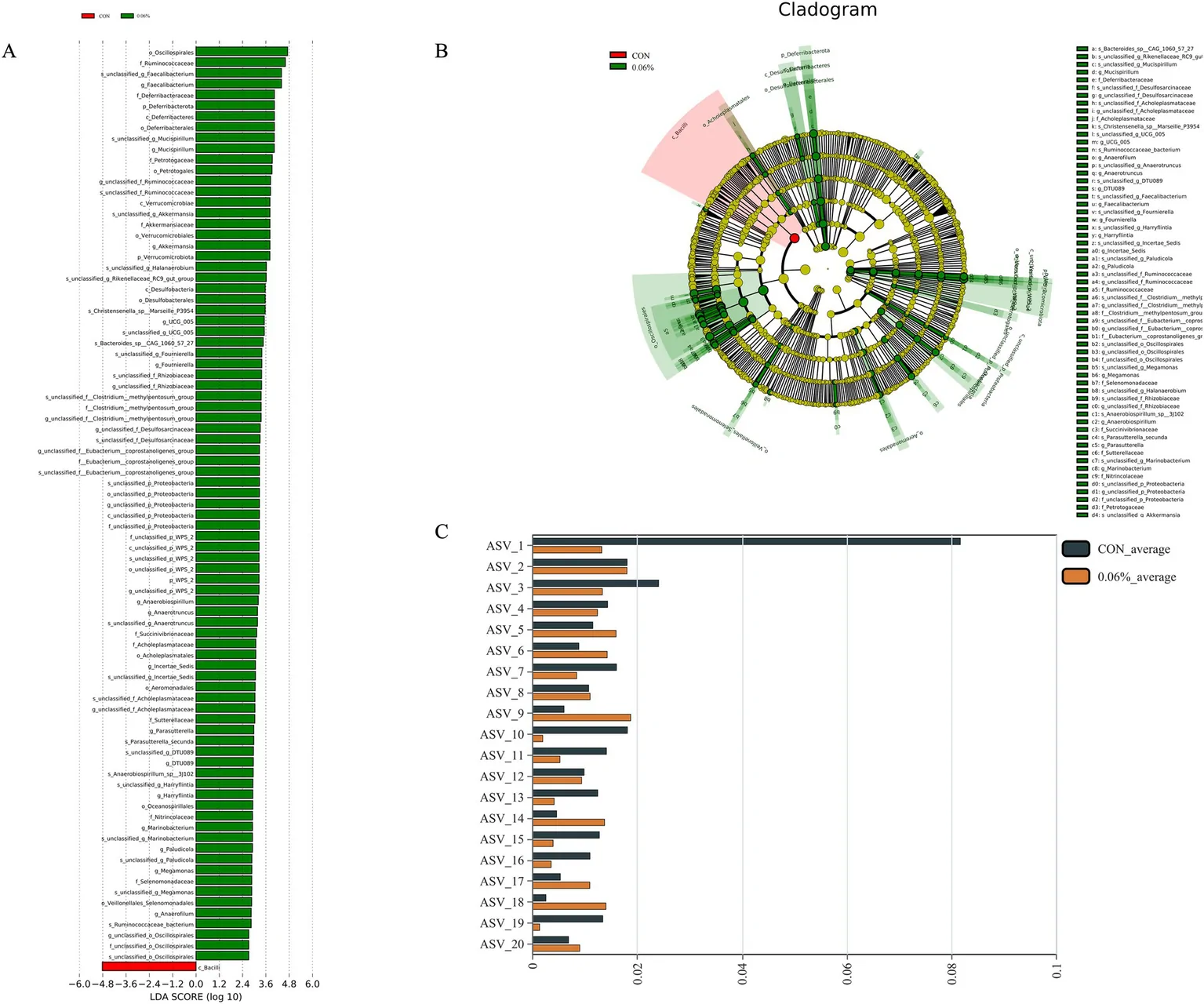
Effects of dietary NCG supplementation on beta diversity of cecal microbiota in Huoyan geese. Principal coordinate analysis (PCoA) based on Bray-Curtis distance was conducted to display the overall structural differences in intestinal microbiota between the blank control (CON) group and the 0.06% NCG supplementation group at phylum **(A)**, family **(B)** and genus **(C)** levels. CON stands for the blank control group, and 0.06% represents the NCG supplementation group.

The evolutionary cladogram from LEfSe analysis (Figure 7B) further visualized the taxonomic distribution of differential microbial taxa. A wide range of taxa within phylum *Firmicutes* were significantly enriched in the 0.06% NCG group, represented by SCFA-producing genera including *Faecalibacterium* and taxa from family *Ruminococcaceae*. By comparison, the control group only showed enrichment of *Lactobacillus*-related taxa belonging to class *Bacilli*. The completely divergent enrichment patterns between the two treatments demonstrated that supplemental 0.06% NCG significantly reshaped the overall cecal microbial community structure of Huoyan geese.

To resolve intergroup microbial differences at the amplicon sequence variant (ASV) level, we systematically analyzed taxonomic annotation, mean relative abundance, and differential characteristics of the top 20 dominant ASVs (Figure 7C). Obvious divergence in ASV abundance profiles was detected between the two groups. ASVs annotated to genus *Lactobacillus* dominated the control group; among them, ASV_1 had an average relative abundance of 8.17% in the control group, far higher than the 1.32% observed in the 0.06% NCG group, making it the most distinct differential ASV. Conversely, the 0.06% NCG group contained elevated abundances of ASVs assigned to SCFA-producing genera such as *Faecalibacterium* and *Blautia*. The overall separation of ASV abundance patterns at the finest ASV resolution corroborated the intergroup microbial differentiation revealed by the LEfSe analysis above.

#### 3.5.4 Alpha diversity analysis of cecal microbiota in Huoyan geese

As shown in Table 5, compared with the control group, the 0.06% NCG group exhibited significantly increased Chao1 and Shannon indices (*p* < 0.05), while the Simpson index was significantly decreased (*p* < 0.05). These results indicated that 0.06% NCG supplementation enhanced microbial species richness, reduced the dominance of specific dominant species, and consequently increased the overall microbial community diversity.

**Table 5:** Statistical significance of cecal microbial α-diversity in Huoyan geese.

Item	Con	0.06%	*P*
Chao1 index	952.33 ± 71.81^b^	1089.33 ± 115.57^a^	0.03
Shannon index	5.29 ± 0.29^b^	5.73 ± 0.12^a^	0.01
Simpson index	0.0213 ± 0.0125^a^	0.0078 ± 0.0016^b^	0.05

Within the same row, values with different lowercase superscripts indicate significant differences (*p* < 0.05), while those with the same or no superscripts indicate no significant differences (*p* > 0.05).

#### 3.5.5 Beta diversity analysis of cecal microbiota in Huoyan geese

As shown in Table 6, to further analyze the differences in gut microbial community structure between the two groups, beta diversity was statistically evaluated using both Adonis and Anosim methods. The results revealed that the difference between the control group and the 0.06% NCG group was extremely significant in the Adonis analysis (*p* < 0.01), and the Anosim analysis also indicated an extremely significant difference in community structure between the two groups (*p* < 0.01), demonstrating that 0.06% NCG supplementation significantly altered the cecal microbial community in Huoyan geese.

**Table 6:** Statistical significance of cecal microbial *β*-diversity in Huoyan geese.

Adonis	Anosim
0.005	0.006

### 3.6 Correlation analysis between cecal microbiota and meat quality

To explore the internal interactions of cecal microbiota and their linkage with meat quality phenotypes in Huoyan geese, we performed Spearman correlation analysis to quantify pairwise associations among microbial taxa at both the phylum (Figure 8A) and genus (Figure 8B) levels via the linkET package. Mantel tests were further conducted to characterize the overall correlation between microbial community composition and meat quality indicators.

**Figure 8:**
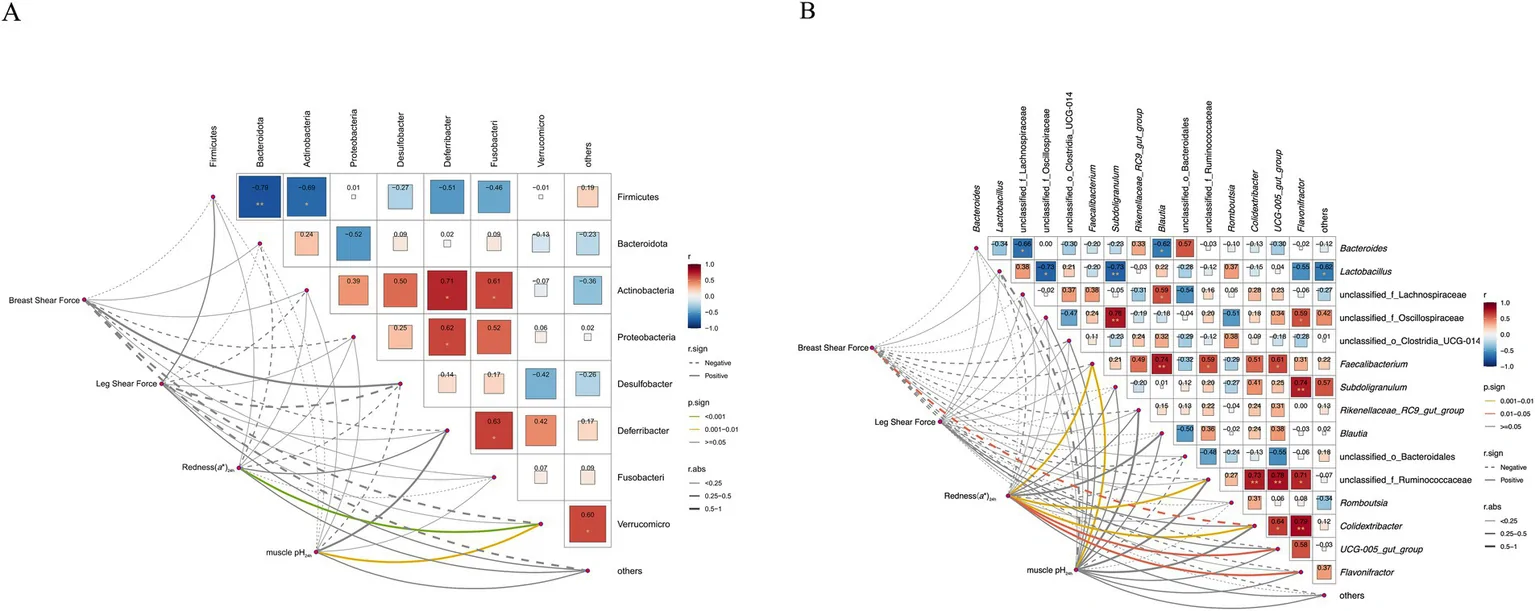
Effects of different dietary NCG doses on correlations between cecal microbiota and meat quality indicators of Huoyan geese. Panel **(A)** shows correlation analysis at the phylum level, and Panel **(B)** shows correlation analysis at the genus level, presenting Spearman correlations between cecal microbiota and meat quality indicators including breast muscle shear force (Shear_breast), thigh muscle shear force (Shear_leg), breast muscle redness (a* at 24 h post-slaughter), and pH value at 24 h post-slaughter (pH24h). In the heatmap, red color indicates positive correlation and blue color indicates negative correlation; ** denotes *p* < 0.01, and * denotes *p* < 0.05. The thickness of connecting lines represents the magnitude of correlation coefficients; solid lines stand for positive correlations, while dashed lines stand for negative correlations.

#### 3.6.1 Phylum-level correlation patterns

Inter-phylum correlation analysis showed Firmicutes had a highly significant negative correlation with Bacteroidota (*r* = −0.79, *p* < 0.01) and a significant negative correlation with Actinobacteria (*r* = −0.69, *p* < 0.05). Actinobacteria had two significant positive correlations with other phyla: Deferribacterota (*r* = 0.71, *p* < 0.05) and Fusobacteriota (*r* = 0.61, *p* < 0.05), while its correlation with Desulfobacterota (*r* = 0.50, *p* > 0.05) was non-significant. Proteobacteriota showed a significant positive correlation with Deferribacterota (*r* = 0.62, *p* < 0.05), but no significant correlation with Fusobacteriota (*r* = 0.52, *p* > 0.05). Verrucomicrobiota was significantly positively associated with unclassified minor phyla (others, *r* = 0.60, *p* < 0.05). All other pairwise phylum combinations exhibited no statistically significant correlations (*p* > 0.05).

Pairwise correlation network analysis between phylum-level microbiota abundance and meat quality traits revealed significant associations for one microbial group. Verrucomicrobiota showed an extremely significant positive correlation with breast muscle redness at 24 h postmortem (redness(a*)24h, *r* = 0.867, *p* < 0.001), indicating higher Verrucomicrobiota abundance may improve breast meat redness. This phylum also had a highly significant positive correlation with postmortem 24 h breast muscle pH (pH 24h, *r* = 0.811, *p* < 0.01). No significant correlation was observed between unclassified minor phyla (“others”) and pH24h (*p* > 0.05). The dominant phyla including Firmicutes, Bacteroidota and Proteobacteriota did not show any significant correlations with breast or leg muscle shear force, or breast muscle redness (*p* > 0.05). At the phylum level, only Desulfobacterota exhibited a significant negative correlation with leg muscle shear force (*r* = −0.58, *p* = 0.05).

#### 3.6.2 Genus-level correlation patterns

At the genus level, *Bacteroides* exhibited significant negative correlations with unclassified_f_*Lachnospiraceae* (*r* = −0.66, *p* < 0.05) and *Blautia* (*r* = −0.62, *p* < 0.05), while its correlation with unclassified_o_*Bacteroidales* (*r* = 0.57, *p* > 0.05) was non-significant. *Lactobacillus* showed highly significant negative associations with unclassified_f_*Oscillospiraceae* (*r* = −0.73, *p* < 0.01), *Subdoligranulum* (*r* = −0.73, *p* < 0.01), as well as a significant negative correlation with unclassified minor genera (others, *r* = −0.62, *p* < 0.05). Unclassified_f_*Lachnospiraceae* was positively correlated with *Blautia* (*r* = 0.59, *p* < 0.05). Unclassified_f_*Oscillospiraceae* possessed highly significant positive correlations with *Subdoligranulum* (*r* = 0.76, *p* < 0.01) and a moderate positive correlation with *Flavonifractor* (*r* = 0.59, *p* < 0.05).

The SCFA-producing genus *Faecalibacterium* showed a highly significant positive correlation with *Blautia* (*r* = 0.74, *p* < 0.01), alongside significant positive correlations with unclassified_f_*Ruminococcaceae* (*r* = 0.59, *p* < 0.05) and UCG-005_gut_group (*r* = 0.61, *p* < 0.05). *Subdoligranulum* was highly positively correlated with *Flavonifractor* (*r* = 0.74, *p* < 0.01). Unclassified_f_*Ruminococcaceae* exhibited extremely significant positive correlations with *Colidextribacter* (*r* = 0.73, *p* < 0.01), UCG-005_gut_group (*r* = 0.78, *p* < 0.01), and *Flavonifractor* (*r* = 0.71, *p* < 0.01). *Colidextribacter* showed a significant positive correlation with UCG-005_gut_group (*r* = 0.64, *p* < 0.05) and a highly significant positive correlation with *Flavonifractor* (*r* = 0.79, *p* < 0.01).

Pairwise correlation network analysis at the genus level further revealed multiple genera significantly associated with meat quality traits. Breast muscle shear force had a significant negative correlation with *Colidextribacter* (*r* = −0.64, *p* < 0.05). Breast muscle redness at 24 h postmortem presented highly significant positive correlations with *Faecalibacterium* (*r* = 0.74, *p* < 0.01), unclassified_f_*Ruminococcaceae* (*r* = 0.79, *p* < 0.01) and *Colidextribacter* (*r* = 0.74, *p* < 0.01), as well as significant positive correlations with UCG-005_gut_group (*r* = 0.62, *p* < 0.05) and *Flavonifractor* (*r* = 0.69, *p* < 0.05). Postmortem 24 h breast muscle pH was highly positively correlated only with *Faecalibacterium* (*r* = 0.80, *p* < 0.01), while no significant correlations were observed between pH24h and unclassified_f_*Ruminococcaceae*, *Colidextribacter*, UCG-005_gut_group or *Flavonifractor* (*p* > 0.05). No genus exhibited significant correlations with leg muscle shear force (*p* > 0.05).

## 4 Discussion

### 4.1 Effects of NCG on growth performance and body size indices of Huoyan geese

In the present study, dietary supplementation with 0.06% NCG significantly reduced the feed-to-gain ratio during the 1–10 weeks of age and increased body weight after 6 weeks of age in Huoyan geese, indicating that appropriate NCG supplementation can improve feed efficiency and growth rate in geese. This finding is consistent with previous reports in broilers (18) and weaned piglets (19). The underlying mechanism may be attributed to the fact that NCG, as an endogenous activator of arginine synthesis, promotes arginine synthesis in intestinal mucosal cells, thereby enhancing the efficiency of protein deposition (20, 21). Arginine is one of the limiting amino acids for growth and development in young animals, and its deficiency can lead to growth retardation. In the present study, geese fed the 0.18% NCG diet exhibited lower body weight after 22 weeks. In the present study, geese fed the 0.18% NCG diet exhibited lower body weight after 22 weeks. This growth depression may arise from unidentified adverse physiological changes. We cannot verify whether metabolic overload or intestinal dysbiosis leads to this phenotype, as microbial samples from the medium and high NCG supplementation groups are unavailable, which is consistent with the growth-inhibitory effects reported in previous high-dose NCG studies (22, 23). Therefore, 0.06% was identified as the optimal supplemental dosage under the conditions of the present study.

Body size indices are important parameters reflecting skeletal development and body conformation. In the present study, the body oblique length, shank length, and shank circumference of Huoyan geese in the 0.06% NCG group were significantly higher than those in the control group, indicating that NCG promotes skeletal development in geese. L-Arginine plays a critical role in regulating growth and development in organisms. It can act directly at the cellular level to promote the gene expression and hormonal secretion of growth hormone (GH) and insulin-like growth factor-1 (IGF-1), thereby activating the hypothalamic–pituitary-somatotropic axis, which constitutes the molecular basis for promoting bone and tissue growth (24, 25). Furthermore, arginine is involved in collagen synthesis and the generation of nitric oxide (NO), which dilates blood vessels and increases blood supply to bone tissues, thereby facilitating osteoblast proliferation and mineralization (26, 27). Therefore, the improving effect of NCG on body size indices may be considered a concomitant manifestation of its overall promotion of growth and development.

### 4.2 Effects of NCG on meat quality

Meat quality is a critical determinant of the economic value and consumer acceptability of poultry meat. In the present study, the 0.06% NCG group exhibited significantly increased breast muscle redness (a*) and pH at 24 h postmortem, along with significantly decreased shear force values in both breast and leg muscles, indicating that NCG supplementation improves meat color, water-holding capacity, and tenderness in geese. The improvement in meat color may be attributed to the ability of NCG to promote myoglobin synthesis or inhibit lipid peroxidation (28). An elevated pH generally indicates a lower degree of postmortem glycolysis and reduced lactate accumulation, which is conducive to improved water-holding capacity and tenderness (29). The reduction in shear force directly reflects an improvement in muscle tenderness, which may be associated with the regulation of calpain system activity or alterations in muscle fiber type composition by NCG (30). Elevated postmortem muscle pH generally reflects a lower degree of muscle glycolysis and less lactic acid accumulation, which is conducive to improving meat water-holding capacity (WHC) and tenderness. Similarly, studies in finishing pigs and growing lambs have demonstrated that arginine supplementation can increase muscle pH and reduce drip loss and shear force (31, 32). Therefore, NCG exerts significant nutritional regulatory effects to improve meat quality in geese.

Intestinal morphology is an important indicator for evaluating intestinal health and nutrient absorption capacity. In the present study, the 0.06% NCG group exhibited significantly increased jejunal villus height and villus height-to-crypt depth ratio, with no significant change in crypt depth, indicating that NCG promoted intestinal epithelial cell proliferation and maturation. The increase in villus height can expand the intestinal absorptive surface area and enhance the utilization efficiency of dietary nutrients, which is consistent with the observed reduction in feed-to-gain ratio in this study. Arginine serves as a critical regulator of intestinal epithelial cell proliferation, promoting protein synthesis and cell growth through the activation of the mechanistic target of rapamycin (mTOR) signaling pathway (33, 34). Furthermore, NCG may reduce intestinal mucosal damage by enhancing local intestinal blood flow and antioxidant capacity (35). Therefore, the improvement in growth performance of geese by NCG can be partly attributed to its positive modulation of intestinal morphology.

### 4.3 Effects of NCG on cecal microbiota

Note that cecal microbiota sequencing was only conducted in the control and 0.06% NCG groups; microbial alterations under 0.12 and 0.18% NCG remain uncharacterized and cannot be discussed herein.

In this study, the effects of NCG on the cecal microbiota of Huoyan geese were first systematically analyzed via 16S rRNA gene sequencing. In terms of microbial community composition, *Firmicutes* and *Bacteroidota* were the predominant phyla in both groups, which are consistent with the typical characteristics of the poultry cecal microbiota (36). Notably, LEfSe analysis and heatmap results revealed that the 0.06% NCG group was significantly enriched with short-chain fatty acid (SCFA)-producing taxa, including *Ruminococcaceae*, *Lachnospiraceae*, *Faecalibacterium*, and *Akkermansia*.

SCFAs, such as butyrate and propionate, serve as important energy substrates for intestinal epithelial cells, providing essential metabolic energy for the renewal and repair of the intestinal mucosa (37), and can significantly inhibit inflammatory responses induced by lipopolysaccharide (LPS) or pro-inflammatory cytokines such as TNF-α (38). Among these taxa, *Faecalibacterium* synthesizes butyrate via the butyryl-CoA: acetate CoA-transferase pathway. It utilizes metabolic intermediates from other microorganisms (e.g., *Bifidobacterium*) as substrates to efficiently produce butyrate through cross-feeding mechanisms, thereby reinforcing intestinal barrier integrity (39). By improving intestinal health and modulating host metabolism, these changes provide compelling indirect evidence for the potential improvement of meat quality. As a mucin-degrading genus colonizing the intestinal mucus layer*, Akkermansia*, has been extensively demonstrated to promote intestinal mucus layer turnover and immune regulation. This bacterium utilizes mucin as a carbon and nitrogen source for its own growth, while simultaneously stimulating the host to produce new mucus, thereby maintaining the dynamic balance of the intestinal barrier (40). In the present study, the abundance of *Lactobacillus* in the control group was significantly higher than that in the NCG group. This phenomenon does not indicate that NCG exerts harmful effects on the gut microbiota, but rather may reflect NCG-induced alterations in ecological niches. When taxa with stronger SCFA-producing capacity, such as butyrate-producing bacteria, become dominant populations within the community, the metabolites they produce (particularly high concentrations of butyrate) and the ecological niches they occupy naturally constrain the relative competitive advantage of Lactobacillus, which shares similar substrate and spatial requirements (41, 42). The reduced abundances of conditionally pathogenic phyla including *Fusobacteriota* and *Desulfobacterota* in the NCG group further confirmed that NCG reshapes the cecal microbiota toward a healthier profile.

### 4.4 Correlation between cecal microbiota and meat quality

In this study, Spearman correlation analysis and Mantel tests further revealed that *Verrucomicrobiota* (including *Akkermansia*) were highly significantly positively correlated with breast muscle redness and pH at 24 h postmortem; *Faecalibacterium*, *Ruminococcaceae*, and *Colidextribacter* also exhibited significant or highly significant positive correlations with breast muscle redness and pH 24 h postmortem. These results strongly suggest that NCG-induced enrichment of beneficial microbiota may represent a potential microbiological mechanism underlying the improvement in meat quality. The possible pathways involved include the following: SCFAs, particularly acetate, propionate, and butyrate, are primarily produced by cecal microbiota through fermentation of dietary fiber. These metabolites not only serve as local energy sources but can also reach distal organs, such as skeletal muscle, via the circulatory system, exerting systemic regulatory effects (43). Multiple lines of evidence have demonstrated that SCFAs can directly influence the glycolytic process and lipid oxidation levels in skeletal muscle (44–46), thereby reducing lactate accumulation and stabilizing pH (47, 48). Furthermore, metabolites of certain gut microbes, such as *Akkermansia*, may influence muscle fiber type transformation through the gut–muscle axis (49).

### 4.5 Limitations of the present study

The present study has certain limitations. First, microbial sequencing was performed solely for the 0.06% NCG group, and the microbial community changes in the 0.12 and 0.18% NCG groups were not analyzed, precluding a comprehensive evaluation of dose-dependent effects. Second, SCFA concentrations in cecal contents were not directly measured, and therefore the inferred functional potential of the microbiota requires further validation through metabolomic analyses. In addition, only female geese were used in this experiment, and the effects of NCG on male geese remain unclear. Future studies should integrate metabolomics, transcriptomics, and gnotobiotic animal models to elucidate the precise molecular mechanisms by which NCG may potentially regulate meat quality through the gut–muscle axis.

## 5 Conclusion

Dietary supplementation with 0.06% N-carbamylglutamate (NCG) significantly improved growth performance (reduced feed-to-gain ratio), enhanced body size indices (body oblique length, shank length, and shank circumference), improved meat quality (increased breast muscle redness and pH at 24 h postmortem, and decreased shear force), and promoted jejunal villus development in Huoyan geese. The underlying mechanism is closely associated with the remodeling of cecal microbial community structure and the enrichment of short-chain fatty acid-producing beneficial bacteria (e.g., *Ruminococcaceae*, *Faecalibacterium*, and *Akkermansia*). Collectively, 0.06% NCG is recommended as the optimal supplemental dosage for improving growth performance and meat quality in Huoyan goose production.

## Data Availability

The data presented in the study is deposited in the https://www.ncbi.nlm.nih.gov/ repository, accession number: PRJNA1492822.
